# MicroRNA-29a Disrupts DNMT3b to Ameliorate Diet-Induced Non-Alcoholic Steatohepatitis in Mice

**DOI:** 10.3390/ijms20061499

**Published:** 2019-03-26

**Authors:** Ya-Ling Yang, Hsing-Chun Kuo, Feng-Sheng Wang, Ying-Hsien Huang

**Affiliations:** 1 Department of Anesthesiology, Kaohsiung Chang Gung Memorial Hospital and Chang Gung University College of Medicine, Kaohsiung 833, Taiwan; yaling453@yahoo.com.tw; 2 Department of Nursing, Chang Gung University of Science and Technology, Chiayi 600, Taiwan; guscsi@gmail.com; 3 Reseach Fellow, Chiayi Chang Gung Memorial Hospital, Chiayi 600, Taiwan; 4 Research Center for the Industry of Human Ecology and Research Center for Chinese Herbal Medicine, College of Human Ecology, Chang Gung University of Science and Technology, Taoyuan 330, Taiwan; 5 Chronic Diseases and Health Promotion Research Center, CGUST, Chiayi 600, Taiwan; 6 Core Laboratory for Phenomics & Diagnostics, Department of Medical Research, Kaohsiung Chang Gung Memorial Hospital and Chang Gung University College of Medicine, Kaohsiung 833, Taiwan; wangfs@ms33.hinet.net; 7 Department of Pediatrics, Kaohsiung Chang Gung Memorial Hospital and Chang Gung University College of Medicine, Kaohsiung 833, Taiwan

**Keywords:** miR-29a, methionine-choline-deficient (MCD) diet, DNMT3b, non-alcoholic steatohepatitis, fibrosis

## Abstract

MicroRNA-29 (miR-29) has been found to reduce liver inflammation and fibrosis following a liver injury. Meanwhile, DNA methyltransferase has been reported to participate in the development of non-alcoholic steatohepatitis (NASH). The aim of this study is to investigate the miR-29a regulation of methyltransferase signaling and epigenetic program in NASH progression. Methods: miR-29a transgenic mice (miR-29aTg mice) and wild-type littermates were subjected to the methionine-choline-deficient (MCD) diet-induced animal model of NASH. Primary hepatic stellate cells were transfected with a miR-29a mimic and antisense inhibitor. We then analyzed gene expressions with qRT-PCR, immunohistochemical stain, Western blot, and luciferase reporter assay. The results demonstrated that increased miR-29a alleviated the MCD diet-induced body weight loss and steatosis and decreased aspartate aminotransferase (AST) levels in mice. Furthermore, hepatic tissue in miR-29aTg mice displayed a weak fibrotic matrix, as shown with Sirius Red staining concomitant with low fibrotic α-SMA expression within affected tissues compared to the wild-type mice fed the MCD diet. Forced miR-29a expression reduced the MCD diet exaggeration of reactive oxygen species (ROS) production by immunohistochemically staining 8-OHdG. Increased miR-29a signaling also resulted in the downregulation of DNMT3b, TGF-β, IL-6, heme oxygenase-1 (HO-1), p-SMAD3, PI3K, and L3BII expression within the liver tissue. An in vitro luciferase reporter assay further confirmed that miR-29a mimic transfection reduced DNMT3b expression in primary HSCs. Our data provide new insights that miR-29a improves MCD diet-induced liver inflammation, steatosis and fibrosis, and highlight the potential of miR-29a targeted therapy for treating NASH.

## 1. Introduction

Non-alcoholic fatty liver disease (NAFLD) occurs when fat is deposited (steatosis) in the liver due to causes besides excessive alcohol use [1] and is one of the most important causes of chronic liver disease. With most studies estimating a prevalence ranging from 25% to 45%, NAFLD increases with the rate of both obesity and diabetes [1,2]. Growing evidence has shown that NAFLD is a major factor in the pathogenesis of insulin resistance, type 2 diabetes, and metabolic syndrome [1], and suggests that diabetes can accelerate NAFLD and liver fibrosis [3]. Non-alcoholic steatohepatitis (NASH), the most extreme form of NAFLD, is considered a key predisposing factor for cirrhosis and has been implicated in the risk of non-viral hepatitis related hepatocellular carcinoma (HCC). Furthermore, it is the most rapidly increasing indication for liver transplantation [1,4]. However, the role of hepatic steatosis in the pathogenesis of NASH and liver fibrosis remains unknown, and few therapeutic agents have yet to improve the liver histology or hepatic protein synthetic function in NASH animal models.

Mice fed methionine-choline-deficient diets (MCD) not only develop fatty liver, but also quickly develop liver fibrosis and hepatocarcinoma [5]. The mechanism of MCD diets responsible for developing fatty liver is associated with the increased synthesis of very-low-density lipoprotein (VLDL) [6] and the subsequently increased cholesterol deposited in the liver. Choline and methionine are dietary methyl donors responsible for maintaining liver function. Previous evidence has demonstrated that rodents given MCD diets have undermethylated DNA methyltransferase genes and thus overexpress these methyltransferases, which explains why these genes are over-methylated despite the methyl-donor deficiency [7]. Controlling epigenetic activity with a DNA methylation inhibitor moderates hepatic wound healing and fibrogenesis [8,9]. In summary, the fibrosis process in the MCD diet animal model is initiated by activating HSC via proinflammatory cytokines and TGF-β signaling, as well as a new set of epigenetic marks that transform HSC.

MicroRNAs (miRNAs) are ~22-nucleotide single-stranded non-coding RNAs (guide strands) that reduce endogenous mRNA transcripts [10]. Interestingly, recent studies have demonstrated that miRNAs have important functions in the pathogenesis of steatosis [11] and their epigenetic regulation, which has been shown to coordinate many aspects of fibrogenesis in the liver [12]. In our previous studies [13,14,15,16,17,18,19], we have already demonstrated that miR-29a overexpression in cholestatic mice significantly inhibited hepatocellular damage and liver fibrosis. Increased miR-29a function inhibits DNA methyltransferases signaling, thus hindering HSC activation [17]. In addition, elevated miR-29a in SMMC-7721 cells, a steatosis hepatic cell model, has been shown to significantly decrease the free cholesterol accumulation in the cells [20]. Furthermore, Jampoka et al. have found that the serum miR-29a levels were significantly lower in NAFLD patients than in the controls [21]. Therefore, in this study, we used miR-29a transgenic mice (miR-29aTg mice) to test whether miR-29a altered the methyltransferase signaling in diet-induced non-alcoholic steatohepatitis in mice.

## 2. Results

### 2.1. Overexpression of miR-29a Significantly Reduced Hepatocellular Steatosis, Fibrosis, and Damage in MCD Diet Mice

**Figure 1:**
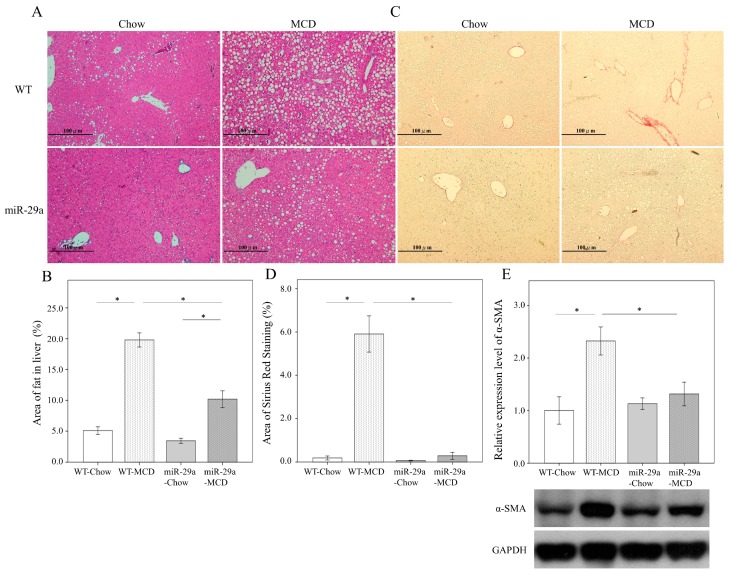
Overexpression of miR-29a significantly reduced hepatocellular steatosis and fibrosis in methionine-choline-deficient (MCD) diet mice. The hematoxylin and eosin stain showed an abundance of fat droplets accumulated in the liver after feeding WT mice the MCD diet for 4 weeks (**A**,**B**, *p* < 0.001). Compared to the WT littermates, miR-29a overexpression reduced the abundance of fat droplets accumulated in the livers of miR-29aTg mice (**A**,**B**, *p* < 0.001). Furthermore, Sirius red stain and western blot demonstrated a greater accumulation of extracellular matrix (**C**,**D**) and α-SMA expression (**E**) in the livers of WT-MCD mice compared to WT-Chou (both *p* < 0.001). However, miR-29a-MCD mice showed a weaker induction of collagenous matrices and α-SMA compared to WT-MCD mice (*p* < 0.001 and *p* = 0.024, respectively). Data are expressed as the mean ± SD of six to eight samples per group. * indicates a *p* < 0.05 between the groups.

To gain insight into the possible involvement of miR-29a in the development of NASH, we used the MCD diet animal model, which has been shown to induce steatosis, hepatic inflammation, and perisinusoidal fibrosis in the liver [22]. As shown in Figure 1A,B, hematoxylin and eosin stain showed an abundance of fat droplets accumulated in the liver after feeding WT mice the MCD diet for 4 weeks (*p* < 0.001). Compared to the WT littermates, miR-29a overexpression reduced the abundance of fat droplets accumulated in the liver of the miR-29aTg mice (Figure 1A,B, *p* < 0.001). Furthermore, Sirius red stain and western blot also showed a greater accumulation of extracellular matrix (Figure 1C,D) and α-SMA expression (Figure 1E) in the livers of WT-MCD mice compared to WT-Chou (both *p* < 0.001). However, the miR-29a-MCD mice also had a weaker induction of collagenous matrices and α-SMA compared to WT-MCD mice (*p* < 0.001 and *p* = 0.024, respectively). Our results indicated that mice given the MCD diet also had significantly increased aspartate transaminase (AST) and alanine transaminase (ALT) levels in the miR-29aTg mice and WT littermates (all *p* < 0.001, Table 1). The overexpression of miR-29a significantly reduced AST levels in the miR-29aTg mice compared with the WT littermates in the MCD diet group. However, we observed no statistically significant difference in ALT levels between miR-29aTg mice and WT littermates in the MCD diet (*p* = 0.983). Collectively, it suggests that the gain of function of miR-29a has a protective effect against MCD diet-induced NASH.

### 2.2. Increased miR-29a Reduced Oxidative Stress in MCD Diet Mice

**Figure 2:**
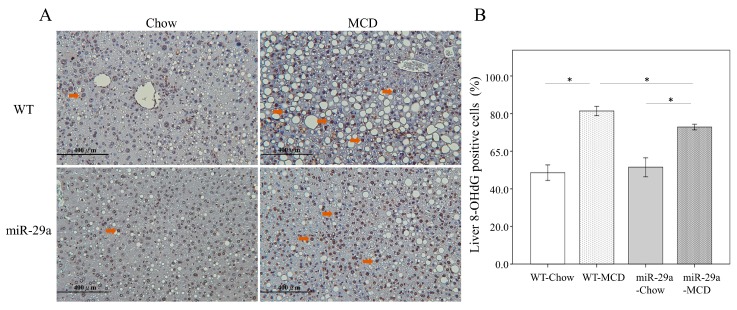
Overexpression miR-29a reduced oxidative stress in MCD diet mice. (**A**)To detect oxidative stress in livers, we used immunohistochemical staining of 8-OHdG. The WT-MCD group exhibited an increased expression of 8-OHdG (arrowhead) compared to the WT-Chou group (**B**, *p* < 0.001). In the miR-29a-MCD group, the abundance of 8-OHdG was significantly lower than in the WT-MCD group (**B**, *p* < 0.001), which indicates the inhibition of oxidative stress to miR-29a signaling in MCD diet-induced NASH. Data are expressed as the mean ± SD of six to eight samples per group. * indicates a *p* < 0.05 between the groups.

To detect oxidative stress in the liver, we immunohistochemically stained 8-OHdG. As demonstrated in Figure 2A,B, the WT-MCD group exhibited an increased expression of 8-OHdG (arrowhead) compared to the WT-Chou group (*p* < 0.001). In the miR-29a-MCD group, the abundance of 8-OHdG were significantly lower than the WT-MCD group (*p* < 0.001), which suggested that oxidative stress was inhibited to miR-29a signaling in the MCD diet-induced NASH.

### 2.3. Overexpression of miR-29a Reduced DNA Methyltransferases in MCD Diet Mice

**Figure 3:**
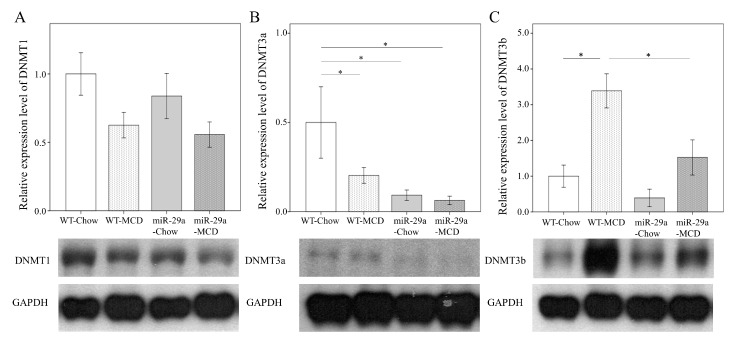
Overexpression of miR-29a reduced DNA methyltransferases in MCD diet mice. Comparison of the activation of DNA methyltransferase 1 (**A**), 3a (**B**), and 3b (**C**) in WT and miR-29Tg mice livers. Data from the six to eight samples per group are expressed as mean ± SD. * indicates a *p* < 0.05 between the groups.

We further examined whether the MCD diet altered the concentrations of DNA methyltransferase protein in the hepatic tissue. As shown in Figure 3, the WT-MCD group exhibited increased DNMNT3b protein levels when compared to the WT-Chou group (*p* < 0.001, respectively). In the miR-29a-MCD group, the abundance of DMNT3b was significantly lower than in the WT-MCD group (*p* = 0.003, respectively), which indicates an active response of DNMT 3b to miR-29a signaling in MCD diet-induced NASH.

### 2.4. miR-29a Overexpression Reduces Cytokines and Autophagy in MCD-Diet Liver

**Figure 4:**
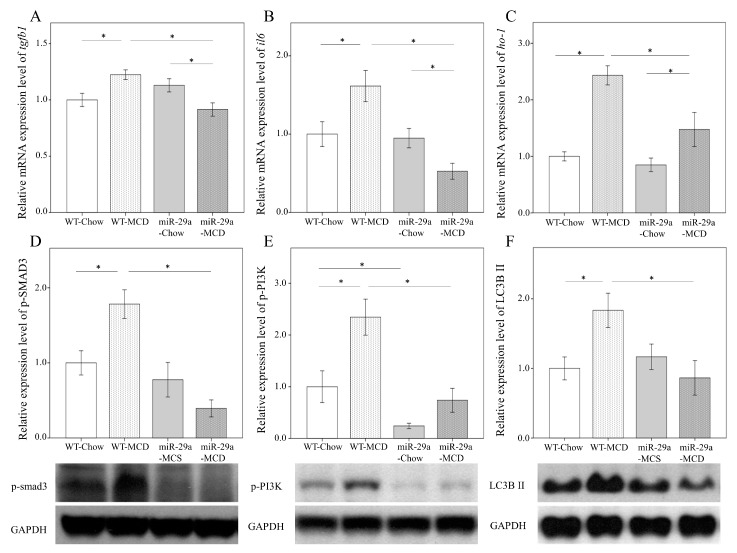
Overexpression of miR-29a reduces cytokines and autophagy in MCD-diet mice. Comparison of the mRNA expression of TGF-β1 (**A**), IL-6 (**B**), and HO-1 (**C**), as well as the protein expression of p-SMAD3 (**D**), p-PI3K (**E**), and LC3BII (**F**) in WT and miR-29Tg mice livers. Data from six to eight samples per group are expressed as mean ± SD. * indicates a *p* < 0.05 between the groups.

We tested whether miR-29a could attenuate proinflammatory cytokine expressions in MCD-diet livers. TGF-β and IL-6 are vital for liver fibrogenesis and inflammation [23,24], and HO-1 is a stress-inducible hepatic antioxidant gene in the MCD-induced NASH animal model [25]. qRT-PCR analyses revealed that the MCD-miR29Tg group exhibited a significant decrease in *tgfb1, il6*, and *ho-1* transcripts (*p* < 0.001, Figure 4A; *p* < 0.001, Figure 4B; *p* = 0.002, Figure 4C, respectively). Both pSMAD3, a transcriptional activator of TGF-β, and p-PI3K have crucial functions in the early activation of HSC [26,27]. Meanwhile, elevated LC3B Ⅱ expression is an important autophagosomal marker [28]. Using western blot analyses, our study revealed that the miR29-MCD group exhibited a significant decrease in p-SMAD3, p-PI3K, and LC3B II protein levels compared with the WT-MCD group (*p* < 0.001, Figure 4D; *p* < 0.001, Figure 4E; *p* = 0.005, Figure 4F, respectively). These data suggest that the hepatoprotective action of miR-29a in MCD-fed mice is the result of the decrease in ROS generation, profibrotic gene expressions, and autophagy.

### 2.5. miR-29a Targeted the 3′-UTR of DNMT3b

**Figure 5:**
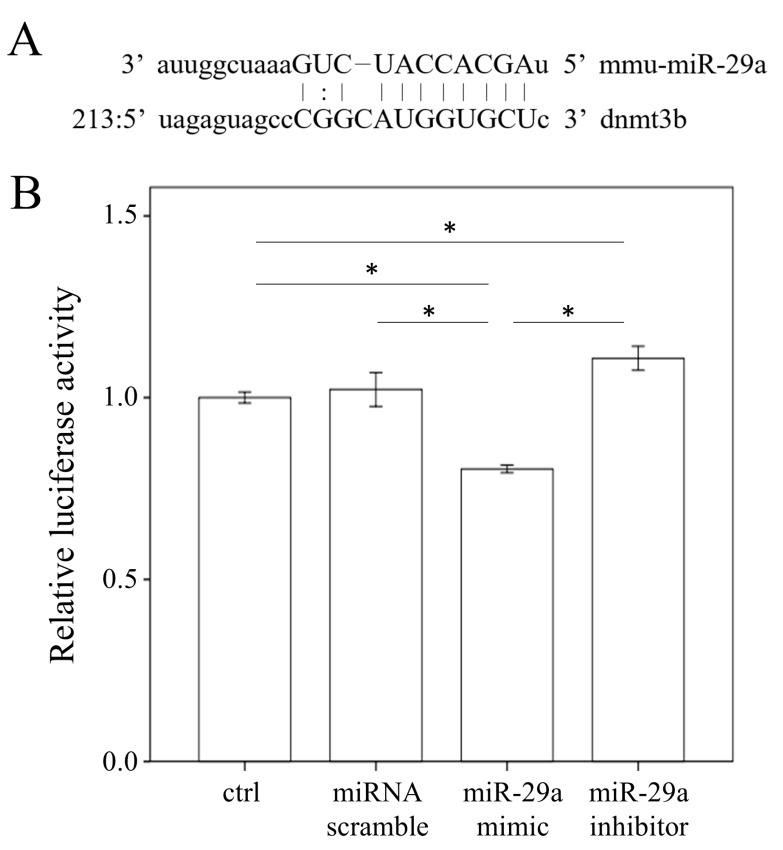
DMNT3b is a direct target of miR-29a. (**A**) Bioinformatic prediction indicates that the seed sequence of miR-29a has a high level of complement to DMNT3b 3′UTR. (**B**) miR-29a mimic transfection into primary hepatic stellate cells inhibited the activity of a luciferase reporter gene linked to the 3′UTR of DMNT3b. Data from six to eight samples per group are expressed as mean ± SD. * indicates a *p* < 0.05 between the groups.

With bioinformatics (www.mirbase.org) predicting that miR-29a will target DNMT3b expression, we hypothesized that miR-29a may directly affect DNMT3b mRNA expression in HSCs (Figure 5A). As shown in Figure 5B, increasing miR-29a expression significantly reduced the 3′-UTR luciferase reporter activity of DNMT3b, while decreasing it reversed this effect (all *p* < 0.001).

## 3. Discussion

The histological spectrum of non-alcoholic steatohepatitis (NASH) is one of the most commonly occurring chronic liver disease. It ranges from hepatic steatosis to steatohepatitis and fibrosis in the digestive tract and is a risk factor for various metabolic diseases, including obesity, type 2 diabetes, and dyslipidaemia [29,30,31]. The primary treatment for NASH involves not only reversing the accumulation of Triglycerides (TG) or alanine transaminase (ALT) or aspartate transaminase (AST) in hepatocytes but also effectively curbing hepatitis, thus preventing steatosis from developing into NASH and fibrosis [31]. However, examining how pharmacological compounds prevent NASH syndrome and understanding the mechanisms underlying NASH pathogenesis is critical. Growing evidence in recent years has shown that increased miR-29 significantly restrains human and murine liver fibrosis and the activation of hepatic stellate cells, while its downregulation affects HSC activation [13,14,15,16,17,18]. In this study, we found that the novel functions of miR-29 on MCD diet include reducing liver steatohepatitis, inflammation, and fibrosis in the livers of miR-29aTg mice. Furthermore, our 8-OHdG study suggested that increased miR-29a results in the inhibition of oxidative stress NASH syndrome in MCD diet. Moreover, the present in vivo study demonstrated that miR-29a-MCD diet livers significantly reduced the expression of TGF-β, IL-6, and HO-1 transcripts, as well as p-SMAD3, p-PI3K, and LC3BII, which represented the inhibition of liver inflammation, fibrosis, and autophagy.

The animal model of MCD diet better mimicked the pathological findings of human NASH than did other dietary models [32]. Inflammation, fibrosis and hepatocellular apoptosis developed much more severely and quickly than in mice fed high fat or Western diets [32]. Thus, the MCD diet also better models for study the mechanisms implicated in the pathogenesis of human NASH or therapy testing. However, the MCD model is limited, because it has known disparities with the metabolic profile of human NASH. Instead of being obese, mice fed an MCD diet show significant body weight loss, cachexia and low triglyceride level [33]. Previous studies have shown that long-term administration of a methionine-choline-deficient diets (MCD) lacking methyl donors caused global DNA hypermethylation, which results in fatty liver and fibrosis [7,34]. MCD diet is also an important risk factor since methyl-deficient diets that alter DNMT3b expression may contribute to hypermethylation in specific areas [35,36]. In our study, we have shown significantly decreased DNMT3b levels in the miR-29a-MCD diet group. Our current in vitro study has consistently demonstrated that miR-29a mimic can act as a novel epigenetic regulator by targeting DNMT3b transcript expression in HSCs cells using pMIR-DNMT3b-29a Luciferase reporter activity. We already know that de novo methylation is carried out through DNMT 3a and 3b [37]. Interestingly, Yang et al. have uncovered that DNMT3b regulates macrophage polarization and inflammation in adipose tissue macrophages [38]. Through epigenetic mechanisms, the elevated saturated fatty acids resulted in increased DNMT3b bound to the promoter region of PPARγ1, which may contribute to deregulated adipose tissue macrophage polarization and ultimately cause inflammation and insulin resistance [38]. Therefore, determining the mechanism of active responses of DNMT3b to miR-29a signaling in MCD diet-inhibited NASH syndrome is critical.

Mounting evidence has indicated that mitochondrial dysfunction resulting from an excess of ROS can alter calcium homeostasis and protein, while the disruption of endoplasmic reticulum (ER) oxidative stress, which is often known as ER stress or UPR, was found in the livers of patients suffering from NASH and obesity [39]. Meanwhile, recent studies have indicated that autophagy is a vital regulatory pathway in liver fibrosis [40] and has been correlated with a direct contribution to HSC activation [41]. Many studies have proposed oxidative stress as one of the most important factors in NASH progression, including hepatocyte apoptosis and the activation of nonparenchymal Kupffer cell and HSCs [42,43]. The obvious effects of miR-29a-MCD diet livers on reducing hepatic p-SMAD3, p-PI3K, and LC3BII, as well as the expression of the oxidative stress-related abundance of 8-OHdG, seem to be in the attenuation of oxidative stress, hepatocyte inflammation, autophagy, and fibrosis. Recent studies have shown that levels of mTOR significantly restrains human and murine adipogenesis and activation of energy metabolism, as previously determined in human NAFLD, using different adipose tissues, while their downregulation obesity and lipotoxicity [44]. Consistently, it was demonstrated that oleuropein, a plentiful phenolic compound, could alleviate high fat diet-induced hepatosteatosis by targeting autophagy signaling pathway [45]. Here, it found that the novel functions of miR-29 on the phosphorylation of AKT/mTOR indicating control hepatocellular carcinoma cell proliferation via the overexpression of miR-29a [46]. Future studies are required that should focus on the link of miR-29a with mTOR pathway can therefore reflect the changes in gain of miR-29a reversed MCD diet hepatic steatosis.

## 4. Materials and Methods

### 4.1. Ethics Statement

Our animal protocol has been reviewed and approved by the Institutional Animal Care and Use Committee (IACUC) of Chang Gung Memorial Hospital (#20161214009). We purchased C57BL/6 mice weighing 25–35 g from BioLASCO Taiwan Co., Ltd. (Taipei, Taiwan) All animals were housed in an animal facility at 22 °C, with a relative humidity of 55%, in a 12 h light/12 h dark cycle, with food and sterile tap water available ad libitum.

### 4.2. Construction and Breeding of the miR-29a Transgenic Mouse Colony

Transgenic mice with overexpressed miR-29a driven by the phosphoglycerate kinase 1 promoter were bred and housed in a specific pathogen-free rodent barrier, as previously described in another study [1]. The genotype of the transgenic mice was probed with PCR and primers (forward: 5′-GAGGATCCCCTCAAGGAT ACCAAGGGATGAAT-3′ and reverse 5′-CTTCTAGAAGGAGTGTTTCTAGGTATCCGTCA-3′). We obtained the wild-type mice from littermates that did not carry the construct.

### 4.3. Animal Model and Experimental Protocol

Six to eight mice were used for all experiments. The mice were categorized into either the “Chou diet” group or the “MCD diet” group. In the NASH mice model, eight-week-old male C57BL/6J mice (CLEA Japan, Tokyo, Japan) were fed an MCS diet (control diet, A02082003B, OPENSOURCE) and MCD diet (A02082002B, OPENSOURCE) for 4 weeks, respectively. The mice’s body weight was recorded daily. Liver tissues were dissected, snap-frozen, and processed to isolate the total RNA and proteins. All specimens were stored at −80 °C until biochemical analysis.

### 4.4. Histological Analysis

For morphometric studies, liver tissues were preserved in 10% formaldehyde, embedded in paraffin, and cut into 3-µm thick sections stained with hematoxylin-eosin or Sirius red. The size of the fat droplets was assessed through hematoxylin and eosin stain, while liver fibrosis was histologically assessed by quantifying the Sirius red–positive area on 10 low-power (magnification, ×40) fields per slide as described in a previous study [47].

### 4.5. 8-OHdG (8-Hydroxy-2′-deoxyguanosine)

To detect oxidative stress in the liver, we adopted the immunohistochemical staining of 8-OHdG. The 10% paraffin-embedded tissues were cut into 3-µm thick sections. After deparaffinization and rehydration, the sections were heated in a citrate buffer (10 mM, pH 6, Thermo Fisher Scientific, Waltham, MA, USA) in a microwave for 30 min to retrieve the antigens. After washing with PBS, all sections were stained using the UltraVision Quanto Detection System HRP DAB kit (Thermo Fisher Scientific, Chino, CA, USA) and hybridized with the anti-8-OHdG antibody (JaICA, FSZ, Tokyo, Japan). Then the sections were counterstained with Mayer’s hematoxylin (ScyTek Laboratories, UT, USA), dehydrated, and mounted using a mounting medium according to the manufacturer’s instructions. The quantification data used image J to count the number of cells and calculate the percentage of dying brown cells.

### 4.6. Real-Time RT-PCR

We used the total RNA (2 µg) extracted from the HSC cells (1 × 10^6^) or liver tissue to generate cDNA with an oligodeoxynucleotide primer (oligo dT15) pursuant to the transcription protocol (Promega, Madison, WI, USA). We followed the manufacturer’s instructions to isolate total microRNA using MicroRNA Isolation Kits (BioChain Institute, Inc., Hayward, Newark, CA, USA). Quantitative RT-PCR between both groups were carried out for *tgfb1*, *il6,* and heme oxygenase-1 (*ho-l*) in the liver. β-actin and sno-202 gene expressions were used to regulate gene and microRNA expression, respectively. qPCR was performed in 10 μL SYBR Green PCR Master Mix (Roche, Basel, Switzerland) containing 10 mM forward primers and reverse primers and approximately 30 ng cDNA. The relative quantification of gene expression was based on the comparative CT method, in which the number of targets was given by 2-(△CT target-△CT calibrator) or 2^-△△Ct^. The primer sequences for some of the representative signaling molecules were as follows: tgfβ1: Forward sequence 5′-ATCCTGTCCAAACTAAGGCTCG-3, Reverse sequence 5′-GACCTCTTTAGCATAGTAGTCCGC-3′; il6: Forward sequence 5′-TTTCCTCTGGTCTTCTGGAGTA-3′, Reverse sequence 5’-CTCTGAAGGACTCTGGCTTTG-3′; heme oxygenase-1(ho-1): Forward sequence 5′-GCCGAGAATGCTGAGTTCAT-3′, Reverse sequence 5′-CTGCTTGTTGCGCTCTATCT-3′.

### 4.7. Western Blotting

The 30-µg protein extracts were mixed with a sample buffer, boiled for 10 min, and followed by electrophoresis using 8–15% sodium dodecyl sulfate-polyacrylamide gels. We transferred the proteins in the gels to a polyvinylidene difluoride membrane and incubated the blots with primary antibodies against, α-SMA (abcam, JHY, Cambridge, UK), DNMT1 (Santa Cruz, CA, USA), DNMT3a (Santa Cruz, CA, USA), DNMT3b (Santa Cruz, CA, USA), p-PI3K (PROTEINTECH, Rosemont, IL, USA), p-SMAD3 (abcam, JHY, Cambridge, UK), LC3B II (Cell signaling, Danvers, MA, USA), and GAPDH (PROTEINTECH, IL, USA) for protein control. After washing the blots with tris-buffered saline and incubating them with horseradish peroxidase-coupled anti-rabbit immunoglobulin-G antibodies (dilution, 1:5000), HRP anti-mouse immunoglobulin-G antibodies (dilution, 1:10,000), and HRP anti-goat immunoglobulin-G antibodies (dilution, 1:10,000) at room temperature for 1 h, we developed them with enhanced chemiluminescence detection (GE Healthcare Biosciences AB, Uppsala, Sweden), exposed them to film, and quantified the signals using densitometry.

### 4.8. Primary HSC Isolation and Culture

Primary HSCs were isolated from the C57BL/6 livers with sequential digestion of the liver with pronase and collagenase, followed by density gradient centrifugation in 8.5% Nycodenz (Sigma-Aldrich, St. Louis, MO, USA) as previously described in another one of our studies [14]. In short, cells were maintained in Dulbecco’s Modified Eagle’s medium (DMEM; Gibco, Thermo Fisher Scientific, Inc., Waltham, MA, USA) with 10% fetal bovine serum. The HSCs had a quiescent phenotype after spending 1 day in culture and developed an activated phenotype after 7–14 days. We carried out the passage of the cultured cells after they reached confluence and performed our experiments using cells between passages 8 and 10.

### 4.9. RNAi Transfection

We used the primary HSC isolation and culture methods that were previously described [47]. Primary HSCs were seeded into 6-cm dishes (9 × 10^5^ cells/dish) overnight and then transfected with a miR-29a precursor (a miR-29a mimic, GE Healthcare Dharmacon, Inc., Lafayette, CO, USA), miR-29a antisense oligonucleotide (GE Healthcare Dharmacon, Inc., Lafayette, CO, USA), or miR control (GE Healthcare Dharmacon, Inc., Lafayette, CO, USA) for 24 h using the Lipofectamine™ RNAiMAX Transfection Reagent (Invitrogen, Carlsbad, CA, USA) in accordance with the manufacturer’s instructions [27].

### 4.10. Luciferase Reporter Assay

With bioinformatics (www.mirbase.org) predicting that miR-29a would target DNMT3b expression, we hypothesized that miR-29a may also directly affect DNMT3b mRNA expression in HSCs. The sequences were annealed and cloned into the pMIR-ReportTM Luciferase plasmid (Applied Biosystems, Foster City, CA, USA) to generate the pMIR-DNMT3b-29a vector in accordance with the manufacturer’s instructions. DNMT3b-3′UTR for miR-29a (position 228–234): forward, 5′-CACCTGTCCCCTTCCTTAGC-3′; reverse, 5′-ACATTCGCAAAA GCGTGCTC-3′. The plasmids were purified using EasyPrep EndoFree Maxi Plasmid Extraction Kit (BIOTOOLS, Ltd., New Taipei, Taiwan). We used empty-vector pMIR without the inserts as a negative control and pMIR-Report β-gal control plasmid for transfection normalization. Primary HSCs were cultured in 24-well plates and transfected with 800 ng of pMIR-29a or pMIR together with 100 ng of pMIR-β-gal and 20 pmol of miR-29a precursor or miRNC (GenePharma, Suzhou, China). Lipofectamine 2000 was used for transfection. Forty-eight hours after transfection, luciferase and β-gal activity were measured using the Dual-Light System (Applied Biosystems).

### 4.11. Statistical Analysis

All values in the figures and tables are expressed as mean ± standard deviation (SD). Quantitative data were analyzed using one-way analysis of variance when appropriate. We adopted the least significant difference (LSD) test for post-hoc testing when appropriate. Two-sided *p*-values less than 0.05 were considered statistically significant.

## 5. Conclusions

Our results demonstrated that the epigenetic mechanism of miR-29a in the mitigation of liver fibrosis and hepatic inflammation targets DNMT3b and thus hinders ROS production with a decreased expression of proinflammatory cytokines and autophagy in the MCD diet-induced NASH animal model. Analysis of the results presented here highlights the benefit that controlling miR-29a signaling can serve as an innovative strategy for treating NASH.

## Figures and Tables

**Table 1:** Comparison of the changes in the four groups.

	WT-Chow	WT-MCD	miR-29a-Chow	miR-29a-MCD
Body weight (g)	27.79 ± 4.10	14.69 ± 1.72 ^a^	24.40 ± 1.81 ^a^	14.36 ± 0.7 ^b^
Body weight gain (%)	22.63 ± 6.20	−46.40 ± 13.79 ^a^	21.06 ± 4.88	−36.3 ± 6.68 ^b,c^
Liver (g)	1.08 ± 0.21	0.56 ± 0.09 ^a^	0.97 ± 0.21	0.49 ± 0.04 ^b^
Liver/body weight (%)	3.88 ± 0.34	3.79 ± 0.32	4.02 ± 1.02	3.41 ± 0.31
AST (U/L)	73.00 ± 31.00	192.6 ± 59.00 ^a^	65.25 ± 21.41	148.09 ± 36.75 ^b,c^
ALT (U/L)	20.00 ± 10.82	102.6 ± 92.58 ^a^	15.75 ± 5.59	103.09 ± 54.05 ^b^

WT, wild type; MCS, methionine-choline-deficient; Data expressed as mean ± standard deviation; ^a^ *p* < 0.05 versus WT-MCS; ^b^ *p* < 0.05 versus miR-29a-MCS; ^c^ *p* < 0.05 versus WT-MCD.
